# Supplementary material for: Time of urine sampling may influence the association between urine specific gravity and body composition
Source: PLoS One. 2025 Sep 26;20(9):e0333479. doi: 10.1371/journal.pone.0333479 (PMC12468934; doi:10.1371/journal.pone.0333479)
Supplement: Attachment — Submitted filename: USGandBodyCompDraft 5-29-2025 rev.docx [file pone.0333479.s002.docx]

**Title:** Time of urine sampling influences the association between urine specific gravity and body composition

**Authors:** Patrick B. Wilson, PhD, RDN^a^,^,^ Brian K. Ferguson, PhD^b^, & Ian P. Winter, MS, RDN^b^,

^a^Associate Professor of Exercise Science, Human Performance Laboratory, School of Exercise Science, Old Dominion University, Norfolk, VA 23529, USA; 757-683-4783; pbwilson@odu.edu

^b^Department of Health and Human Performance, Palm Beach Atlantic University, West Palm Beach, FL 33401, USA; 561-803-2385; brian_ferguson@pba.edu

^c^Human Performance Laboratory, School of Exercise Science, Old Dominion University, Norfolk, VA 23529, USA; 757-683-4783; iwint001@odu.edu

**ORCID**

Wilson: 0000-0003-4052-5023

**Corresponding Author:** Patrick Wilson, PhD, RDN, Associate Professor of Exercise Science, School of Exercise Science, Old Dominion University, Norfolk, VA 23529, USA; 757-683-4783; pbwilson@odu.edu

**Abstract**

Urine specific gravity (USG) is frequently utilized in practice and research to assess hydration status. Prior research suggests that individuals with large amounts of fat-free mass (FFM) have elevated USG, but little is known about whether the time of collection (first-morning vs. later spot sampling) and various nutritional factors influence the relationships between FFM and USG. This cross-sectional, observational study assessed first-morning (n=55) and spot USG (n=51) samples in adults and evaluated relationships of USG with body composition and nutrition intake. The InBody 770 was used to estimate FFM, skeletal muscle mass (SMM), and total body water (TBW). Protein, fluid, and sodium intakes from the 24-hour period before USG assessments were generated based on the Automated Self-Administered 24-hour Recall (ASA24). Median USG was higher for first-morning samples than spot samples (1.018 vs. 1.011, Z=-5.2, p<0.001). Using a USG threshold of ≥1.020, 41.8% of participants were supposedly hypohydrated in the morning while the prevalence of supposed hypohydration was 21.6% using spot samples. None of the body composition variables (FFM, SMM, TBW) significantly associated with first-morning USG (Spearman ρ<0.10), while all three variables showed significant, positive associations with spot USG (Spearman ρ=0.32-0.36, p<0.05). None of the dietary variables (protein, moisture, sodium) were significantly associated with either first-morning or spot USG. Although previous research has shown the FFM positively associates with USG, this investigation provides evidence that this relationship may depend on sampling time. Spot samples, in comparison to first-morning samples, may be impacted by FFM to a greater degree.

**Keywords**: assessment; fat-free mass; fluid; nutrition; urinary.

**Introduction**

Urine specific gravity (USG) is frequently used in sport and other settings (e.g., research, occupational) to diagnosis hypohydration, often based on a threshold of ≥1.020 [1,2]. Despite the common use of this practice, evidence for using USG on its own as a means of assessing hydration status remains questioned in the literature [3,4]. Previous literature suggests that USG may be more susceptible to misclassifying hydration status when acute body water fluxes occur, such as when active individuals and athletes experience large fluid losses from sweating [3,5].

Another potential—and perhaps underappreciated—problem with using USG to identify hypohydration is that USG may be naturally elevated in individuals with larger body masses [6-9]. In a cross-sectional study of male rugby players and distance runners, Hamouti et al. [6] found significant differences in USG based on body size. The rugby players, who weighed 29 kg more than the runners, had higher average first-morning USG (1.021 vs. 1.016) across six sampling days, and this led to more false positives in assessing hypohydration. A likely reason why larger individuals have elevated USG is that they also have more fat-free mass (FFM) and muscle, body tissues that are major sources of urinary metabolites like creatinine. Baxmann et al. [10], examined a number of urinary metabolites and found stronger correlations between creatinine levels (serum and urine) and lean mass than between creatinine and body weight. In addition, multiple studies have found small-to-moderate-sized positive associations between FFM or skeletal muscle mass (SMM) and USG [6-9]. In totality, current literature tentatively supports the idea that individuals with larger amounts of FFM and muscle may be over-diagnosed with hypohydration when using a set USG threshold of ≥1.020, though this requires confirmation [9].

Beyond body composition, USG can also be affected by dietary intake. Unsurprisingly, While USG shows negative associations with daily fluid intake in the literature [5], other dietary factors may influence USG independently of fluid intake [3]. Dietary protein, for example, seems to affect USG via metabolic byproducts of protein metabolism (e.g., urea) that are excreted via urine [11,12]. As reported by Martin et al. [11], individuals show increased USG when fed high-protein diets. In addition, consumption of electrolytes, particularly sodium, can acutely impact USG [13,14].

Studies have also shown that USG varies based on sampling time of day [3], with first-morning USG values often being higher than spot samples taken later in the afternoon [15]. Whether urine sampling time influences the association between FFM and USG, however, remains unknown. Among studies that have reported a positive correlation between FFM/muscle mass and USG, urine sampling times have varied substantially [6,8,9].

To date, few studies have simultaneously examined the relative contribution of the aforementioned factors (FFM, protein intake, sodium intake, fluid intake) to USG levels. In addition, examining whether these associations differ between first-morning and later-in-the-day spot samples, given the typical variation in USG across the day. Thus, the purpose of the present study was to assess both first-morning and later-in-the-day spot USG samples and their relation to body composition and nutritional intakes, particularly of protein, fluid, and sodium. We hypothesized that FFM, skeletal muscle mass (SMM), dietary protein intake, and dietary sodium intake would associate positively with USG, while fluid intake would negatively associate with USG.

**Methods**

***Participants***

Participants were recruited from a large, mid-Atlantic university and the surrounding community. The recruitment period lasted from December 1, 2021 to August 31, 2024. All participants were at least 18 years of age, weighed under 500 pounds, self-reported engaging in moderate-to-vigorous physical activity at least 3 days per week, and stated they were free from any urinary tract infection. Additionally, due to contraindications for the specific body composition devices used in this study, participants were excluded based on having an implanted electrical device such as a pacemaker or extreme claustrophobia.

After completing eligibility screening, participants attended an initial visit (either via Zoom or in-person) where they were informed about the study’s procedures, risks, benefits, etc., and signed a consent form approved by the Old Dominion University Institutional Review Board. Sixty individuals consented and enrolled to participate in the current study, five individuals did not complete an in-person visit. An additional four participants completed the morning visit but did not complete their later-in-the-day spot sample visit. Thus, the available sample sizes varied based on the visit; n=55 (40 men, 15 women; median age: 26 [22-33] y) for the first-morning visit and n=51 (37 men, 14 women; median age: 26 [22-34] y) for the spot sample visit.

***Procedure****s*

Participants were asked to refrain from vigorous exercise and refrain from food and fluid intake for at least 12 and 8 hours, respectively, prior to the first laboratory visit. After arrival to the laboratory, participants provided a urine sample that was their first void of the day. Each sample was tested twice using a handheld digital refractometer (PAL-10S, Atago, Japan), and the average of the two values was used. Before each test, the refractometer was tested with distilled water and re-zeroed if distilled water was measured at a value different from 1.000. Then, a sucrose standard of 4 g / 100 mL water was measured, with values between 1.016 and 1.018 being deemed acceptable [16].

Upon completion of USG measurement, body composition was measured using air displacement plethysmography (Bod Pod, Life Measurement Instruments, Concord, CA) and bioelectrical impedance (InBody 770, InBody USA, Cerritos, CA). The Bod Pod and InBody 770 both provided estimates of FFM, while the InBody 770 also gave estimates of SMM and total body water (TBW). Although InBody 770 has been shown to overestimate SMM as compared to estimates derived from dual-energy x-ray absorptiometry, the values are highly associated (r > 0.9) [17]. One participant declined to do the Bod Pod test, leaving n=50 for those data. The FFM estimates from the Bod Pod and InBody 770 were highly correlated (Pearson r=0.97) and showed relatively small absolute differences in FFM (1.3 kg); thus, only estimates from the InBody were used for analyses with USG for the sake of reducing risks of type 1 error.

Participants subsequently completed a 24-hour dietary recall utilizing the Automated Self-Administered 24-hour Recall from the National Cancer Institute [18]. This recall tool assists participants in documenting all food and fluid intake over the past 24 h for the estimation of nutrient intakes, including dietary protein, moisture, and sodium. This method provided a reasonably accurate estimation of recent protein intake [19]. Participants were also asked to complete a background questionnaire to include demographic information (age, race/ethnicity, sex).

An additional laboratory visit was conducted on a separate day, typically at 12 p.m. or later, for the measurement of spot USG. Two participants completed this visit at a slightly earlier time (between 11 a.m. and noon). Upon arrival, participants reported the approximate time of their last urine void. Other procedures mirrored those from the prior visit, except for body composition testing, time of urine sampling, and instructions around pre-visit food and fluid intake. Body composition is generally consistent across short time intervals in non-dieting adults [20], so there was no reason to repeat measures. Participants were not required to be in a fasted state or to restrict fluid intake beforehand. Due to technical problems, two participants neglected to complete the 24-h dietary recall at this spot sample visit.

***Statistical analysis***

The data were analyzed with version 29 of SPSS software (IBM Corp., Armonk, NY, USA). The distribution of variables was analyzed by inspecting histograms and Q-Q plots. Age, body fat (kg), spot USG, and dietary intakes associated with the spot USG visit all showed evidence of a right skew. Median (25^th^-75^th^ percentile) was used for descriptive statistics to keep the data presentation uniform across all variables. A Wilcoxon-Signed Ranks test was used to compare USG values between first-morning and later-in-the-day spot samples. Spearman’s ρ was used to evaluate the associations between variables. A two-sided alpha <0.05 was considered as the threshold for statistical significance.

**Results**

Median (25^th^-75^th^ percentile) values for USG were 1.018 (1.014-1.023) and 1.011 (1.003-1.018) for the first-morning and spot samples, respectively. Among the 51 participants with both samples, first-morning USG was higher than spot USG based on a Wilcoxon-Signed Ranks test (Z=-5.2, p <.001). Using a USG threshold of ≥ 1.020, 41.8% of participants were supposedly hypohydrated in the morning while the prevalence of supposed hypohydration was 21.6% using spot samples. There was a modest-sized positive correlation between first-morning and spot USG values (n=51; ρ=0.39, p=.005). There was also a significant positive correlation (n=51; ρ=0.32, p=.021) between later-in-the-day spot USG and time duration since last void (i.e., total hours from last pre-visit void to time of spot USG).

InBody 770 body composition data for the full sample (n=55) were as follows: body mass, 78.9 (67.7-86.7) kg; FFM, 64.2 (54.1-69.9) kg; fat mass, 14.3 (10.2-20.2) kg; SMM, 36.7 (30.0-40.4) kg; and TBW, 47.0 (39.5-51.1) kg. Values were not appreciably different among the 51 participants who completed both visits: body mass, 78.0 (66.1-86.7) kg; FFM, 63.6 (52.1-70.1) kg; fat mass, 14.3 (10.1-20.5) kg; SMM, 36.0 (29.5-40.5) kg; and TBW, 46.6 (38.0-51.2) kg.

Correlations between body composition data and USG are shown in **Table 1**. None of the body composition variables were associated with first-morning USG, while FFM, SMM, and TBW all showed significant, positive associations with spot USG. The relationship between SMM and spot USG values is presented visually in **Figure 1**.

Descriptive data for the nutrition intake data are presented in **Table 2**, with correlations between USG and dietary data shown in **Table 3**. None of the variables of interest (protein, moisture, sodium) were significantly associated with either first-morning or spot USG.

**Discussion**

In agreement with several previous investigations, the present study found that measures of body composition—principally FFM and SMM—were positively associated with USG [6,8,9]. This is the first study, at least to the authors’ knowledge, to show that these relationships may depend on time of sampling. Specifically, later-in-the-day spot samples showed a positive association between FFM/SMM and USG, while first-morning samples did not show a significant association between these variables. Other authors have argued that first-morning urine samples are more valid as a measure of hydration status than spot samples due to minimization of confounding factors (diet, activity, etc.) [3]; the findings of the present investigation provide additional rationale for preferentially using first-morning samples over spot samples when assessing hydration status. Given the observed association between FFM and USG from spot samples, misclassification of hydration status may be more likely when individuals of varying sizes and body compositions have their USG tested after the first void of the day. At a minimum, the results of this and other investigations imply that different USG thresholds should be utilized to identify hypohydration in individuals of different sizes.

Urine sampling times have been variable among prior studies that have found that FFM and SMM are associated with USG. Hamouti et al. [6] evaluated USG upon waking across six days in male rugby players and runners and found a moderate-sized correlation between estimated muscle mass and USG (*r* = 0.50, *p* = 0.03). It is difficult to say why their results for first-morning USG differ from the present study’s findings, however sample size differences may account for these differences. A later analysis by Wilson [8], which found positive associations between FFM and having a USG ≥1.020, was based on spot samples taken at various times throughout the day (morning, afternoon, or evening). Subsequently, Wilson and Winter [9] performed a quantitative review of the literature and reported that FFM had a significant, positive association with USG in both men (n=91 estimates; *ρ* = 0.36, *p* < 0.001) and women (n=22 estimates; *ρ* = 0.57, *p* = 0.006). However, the studies included in their review utilized a variety of sampling approaches to quantify USG (single value vs. average of values; first-morning vs. spot sample), and their analysis was unable to account for those factors. Due to the somewhat inconsistent findings to date, additional research examining the influence of time of day on the connection between FFM and USG is warranted. Time from last void to sample collection should also be examined as a modifying factor given that it showed a positive association (ρ=0.32) with spot USG values in this study.

Our hypotheses related to dietary intakes and USG were not supported by the results. While higher intakes of dietary protein and sodium have been shown to increase USG in some experiments [11,13,14], they did not correlate significantly with either first-morning or spot USG in the present study. Martin et al. [11] revealed that a high-protein diet increased USG relative to a moderate protein diet, but the amount of protein in the high group (3.6 g/kg/day) was well beyond the protein intake in our study, which may partly explain the discrepant findings. Regarding sodium, ingesting large amounts acutely can impact USG, but the effects depend on the volume of fluid ingested alongside sodium, among other factors [13]. In addition, less is known about how chronic high-sodium diets impact USG, though a recent experiment found little effect of a 7-day high-sodium diet [21].

While not observed in all research [1], measures of fluid intake often correlate with USG in various populations (e.g., [5,22]). However, the relationship between fluid intake and USG is likely to depend on several factors, including the method of assessment for each variable. For example, Perrier et al. [5] found that USG values based on 24-hour urine collections were associated with fluid intake while USG from first-morning samples was not. Other specific reasons for the lack of significant association between fluid intake and USG in our study could include the unknown validity of the moisture variable from the Automated Self-Administered 24-hour Recall and our modest sample size. Perrier et al. [5] found a non-significant correlation between first-morning USG and total fluid intake that was relatively similar in size (r = -0.33) to the observed association between first-morning USG and dietary moisture in this study (ρ = -0.21).

There are a few important limitations to this investigation. While the Automated Self-Administered 24-hour Recall is a widely used method to assess recent dietary intake [18], its validity for some nutrients, particularly dietary moisture, is unknown. In addition, while the estimate of SMM from the InBody 770 has shown promising validity [17,23], it requires further validation in a variety of populations. The sample size of this study was not sufficient for detecting small effects (ρ < 0.3), meaning that we cannot rule out the possibility that the dietary variables may have real, albeit minor, associations with USG. Furthermore, we did not directly measure urine metabolites like creatinine, which would have strengthened our argument that the associations between FFM/SMM and USG are likely to be causal in nature.

**Conclusions**

The present study replicates prior literature showing that there are positive associations between FFM/SMM and USG. A novel finding of this work is that the associations between FFM/SMM and USG may vary based on the time of urine sampling, which has implications for the use of USG as a measure of hypohydration in clinical and research settings. Practitioners and researchers should bear in mind that, in contrast to first-morning samples, spot USG samples may depend more on the body size and composition of the individual being evaluated. Additional research should be conducted to confirm that these findings extend to other populations and situations.

**References**

1. Osterberg KL, Horswill CA, Baker LB. Pregame urine specific gravity and fluid intake by National Basketball Association players during competition. J Athl Train. 2009;44(1):53-57
2. Stover EA, Petrie HJ, Passe D, Horswill CA, Murray B, Wildman R. Urine specific gravity in exercisers prior to physical training. Appl Physiol Nutr Metab. 2006;31(3):320-327.
3. Cheuvront SN, Kenefick RW, Zambraski EJ. Spot urine concentrations should not be used for hydration assessment: a methodology review. Int J Sport Nutr Exerc Metab. 2015;25(3):293-297.
4. Cheuvront SN, Muñoz CX, Kenefick RW. The void in using urine concentration to assess population fluid intake adequacy or hydration status. Am J Clin Nutr. 2016;104(3):553-556
5. Perrier E, Rondeau P, Poupin M, Le Bellego L, Armstrong LE, Lang F, Stookey J, Tack I, Vergne S, Klein A. Relation between urinary hydration biomarkers and total fluid intake in healthy adults. Eur J Clin Nutr. 2013;67(9):939-943
6. Hamouti N, Coso JD, Ávila A, Mora-Rodriguez R. Effects of athletes’ muscle mass on urinary markers of hydration status. Eur J Appl Physiol. 2010;109(2):213-219.
7. Ehlert AM, Wilson PB. The associations between body mass index, estimated lean body mass, and urinary hydration markers at the population level. Meas Phys Ed Exer Sci. 2021;25(2):163-170.
8. Wilson PB. Associations of urine specific gravity with body mass index and lean body mass at the population level: Implications for hydration monitoring. Int J Sport Nutr Exerc Metab. 2021;31(6):475-481
9. Wilson PB, Winter IP. Fat‐free mass is positively associated with urine specific gravity in athletes and active adults: a quantitative review. Transl Sports Med. 2024;2024(1):8827027
10. Baxmann AC, Ahmed MS, Marques NC, Menon VB, Pereira AB, Kirsztajn GM, Heilberg IP. Influence of muscle mass and physical activity on serum and urinary creatinine and serum cystatin C. Clin J Am Soc Nephrol. 2008;3:348-354
11. Martin WF, Cerundolo LH, Pikosky MA, Gaine PC, Maresh CM, Armstrong LE, Bolster DR, Rodriguez NR. Effects of dietary protein intake on indexes of hydration. J Am Diet Assoc. 2006;106(4):587-589
12. Miller M, Price JW, Longley LP. Effect of varying intake of protein and salts on the composition and specific gravity of urine. J Clin Investig. 1941;20(1):31-38
13. Savoie FA, Dion T, Asselin A, Goulet ED. Sodium-induced hyperhydration decreases urine output and improves fluid balance compared with glycerol-and water-induced hyperhydration. Appl Physiol Nutr Metab. 2015;40(1):51-58
14. Udokang NE, Akpogomeh BA. Effect of volume loading with water, normal saline, palm wine and lipton tea on urinary output, pH, specific gravity, sodium and potassium concentrations in human subjects. Niger J Physiol Sci. 2005;20(1):101-108
15. Perrier E, Demazières A, Girard N, Pross N, Osbild D, Metzger D, Guelinckx I, Klein A. Circadian variation and responsiveness of hydration biomarkers to changes in daily water intake. Eur J Appl Physiol. 2013;113:2143-2145.
16. Wardenaar F, Ortega-Santos CP, Vento K, Olzinski S, Olig J, Kavouras S, Johnston C. Reliability of 3 urine specific gravity meters for measuring brix and urine solutions at different temperatures. J Athl Train. 2021;56(4):389-395.
17. Lee SY, Ahn S, Kim YJ, Ji MJ, Kim KM, Choi SH, Jang HC, Lim S. Comparison between dual-energy X-ray absorptiometry and bioelectrical impedance analyses for accuracy in measuring whole body muscle mass and appendicular skeletal muscle mass. Nutrients. 2018;10(6):738. doi: 10.3390/nu10060738.
18. Subar AF, Kirkpatrick SI, Mittl B, Zimmerman TP, Thompson FE, Bingley C, Willis G, Islam NG, Baranowski T, McNutt S, Potischman N. The automated self-administered 24-hour dietary recall (ASA24): a resource for researchers, clinicians and educators from the National Cancer Institute. J Acad Nutr Diet. 2012;112(8):1134-1137.
19. Conway JM, Ingwersen LA, Moshfegh AJ. Accuracy of dietary recall using the USDA five-step multiple-pass method in men: an observational validation study. J Am Diet Assoc. 2004;104(4):595-603
20. Anderson DE. Reliability of air displacement plethysmography. J Strength Cond Res. 2007;21(1):169-172
21. McMillan RK, Stock JM, Romberger NT, Wenner MM, Chai SC, Farquhar WB. The impact of dietary sodium and fructose on renal sodium handling and blood pressure in healthy adults. Physiol Rep. 2025;13(6):e70284
22. Heen E, Yassin AA, Madar AA, Romøren M. Estimates of fluid intake, urine output and hydration-levels in women from Somaliland: a cross-sectional study. J Nutr Sci. 2021;10:e66. doi: 10.1017/jns.2021.54
23. Oyama K, Ogawa H, Osaki M, Kono S, Hasegawa M, Izutani Y. Validation of InBody 770 as a tool for assessing skeletal muscle mass. Clin Surg. 2019;4:2637,

| **Table 1** Correlations between USG and body composition data from the InBody 770 | | |
| --- | --- | --- |
|  | First-morning USG (n=55) | Spot USG (n=51) |
| Fat-free mass | 0.08 (.582) | 0.32 (.021)* |
| Fat mass | 0.12 (.386) | -0.13 (.359) |
| Skeletal muscle mass | 0.08 (.551) | 0.36 (.009)** |
| Total body water | 0.07 (.598) | 0.32 (.024)* |
| USG, urine specific gravity. * p<.05, **p<.01. Correlation co-efficients are based on Spearman’s ρ. p values are shown in parentheses. | | |

| **Table 2** Nutrient intakes over the 24-h preceding each visit. | | |
| --- | --- | --- |
|  | **First-morning USG (n=55)** | **Spot USG (n=49)** |
| Energy (kcal) | 2,292 (1,723-2,846) | 2,555 (1,650-3,075) |
| Protein (g) | 115 (76-154) | 148 (85-177) |
| Moisture (g) | 2,661 (1,600-3,831) | 3,008 (1,519-4,721) |
| Sodium (mg) | 4,134 (2,847-5,652) | 3,979 (3,228-6,405) |
|  | | |

| **Table 3** Correlations between USG and dietary intake data | | |
| --- | --- | --- |
|  | **First-morning USG (n=55)** | **Spot USG (n=49)** |
| Protein | -0.21 (.134) | 0.26 (.066) |
| Moisture | -0.21 (.121) | 0.05 (.711) |
| Sodium | -0.17 (.227) | 0.13 (.391) |
| Correlation co-efficients are based on Spearman’s ρ. p values are shown in parentheses. | | |

**Figure 1** – Scatterplot of the relationship between skeletal muscle mass (SMM) and spot sample USG, with line of best fit and 95% confidence intervals shown.

**Supporting Information Caption**

S1 File. Dataset
